# Reaching Mothers and Babies with Early Postnatal Home Visits: The Implementation Realities of Achieving High Coverage in Large-Scale Programs

**DOI:** 10.1371/journal.pone.0068930

**Published:** 2013-07-17

**Authors:** Deborah Sitrin, Tanya Guenther, John Murray, Nanlesta Pilgrim, Sayed Rubayet, Reuben Ligowe, Bhim Pun, Honey Malla, Allisyn Moran

**Affiliations:** 1 Save the Children, Washington, D.C., United States of America; 2 Consultant Medical Epidemiologist, Iowa City, Iowa, United States of America; 3 Johns Hopkins University, Baltimore, Maryland, United States of America; 4 Save the Children, Dhaka, Bangladesh, United States of America; 5 Save the Children, Lilongwe Malawi; 6 Save the Children, Kathmandu, Nepal; Iran University of Medical Sciences, Iran (Republic of Islamic)

## Abstract

**Background:**

Nearly half of births in low-income countries occur without a skilled attendant, and even fewer mothers and babies have postnatal contact with providers who can deliver preventive or curative services that save lives. Community-based maternal and newborn care programs with postnatal home visits have been tested in Bangladesh, Malawi, and Nepal. This paper examines coverage and content of home visits in pilot areas and factors associated with receipt of postnatal visits.

**Methods:**

Using data from cross-sectional surveys of women with live births (Bangladesh 398, Malawi: 900, Nepal: 615), generalized linear models were used to assess the strength of association between three factors - receipt of home visits during pregnancy, birth place, birth notification - and receipt of home visits within three days after birth. Meta-analytic techniques were used to generate pooled relative risks for each factor adjusting for other independent variables, maternal age, and education.

**Findings:**

The proportion of mothers and newborns receiving home visits within three days after birth was 57% in Bangladesh, 11% in Malawi, and 50% in Nepal. Mothers and newborns were more likely to receive a postnatal home visit within three days if the mother received at least one home visit during pregnancy (OR2.18, CI1.46–3.25), the birth occurred outside a facility (OR1.48, CI1.28–1.73), and the mother reported a CHW was notified of the birth (OR2.66, CI1.40–5.08). Checking the cord was the most frequently reported action; most mothers reported at least one action for newborns.

**Conclusions:**

Reaching mothers and babies with home visits during pregnancy and within three days after birth is achievable using existing community health systems if workers are available; linked to communities; and receive training, supplies, and supervision. In all settings, programs must evaluate what community delivery systems can handle and how to best utilize them to improve postnatal care access.

## Introduction

Preventing neonatal deaths is critical to reducing global child mortality rates and achieving Millennium Development Goal (MDG) 4. Deaths within the first month after birth now account for over 40% of all deaths among children under five [1]. Although neonatal mortality declined 32% between 1990 and 2011, it reduced at half the speed of maternal mortality and one-third slower than deaths amongst children aged 1–59 months [1], [2]. Changing the trajectory for neonatal mortality is especially important in high-burden regions of sub-Saharan Africa and South Asia [3].

A landmark 2005 study showed that proven interventions, including preventive and curative services, could avert up to two-thirds of newborn deaths [4]. Yet nearly half of births in low-income countries occur without a skilled attendant, and even fewer mothers and babies have postnatal contact with providers who can deliver interventions that save lives [5].

In 2009, the World Health Organization (WHO) and the United Nations Children’s Fund (UNICEF) released a Joint Statement recommending home visits as a strategy to reduce neonatal deaths [6], based on evidence from research studies demonstrating home visits by trained community-based workers can improve key newborn care practices, care-seeking, and, in high mortality settings, reduce newborn mortality [7], [8], [9], [10], [11]. Early postnatal home visits were emphasized (ideally on days 1, 3, and 7 after birth) because most deaths occur during the first 48 hours after birth and there was evidence from Bangladesh that newborns visited within 2 days were less likely to die [12], [13]. Since the release of the Statement, a growing number of countries have incorporated home-based postnatal visits into child health strategies, and community health workers have been tasked with this effort [5]. More recently, the WHO-led Partnership for Maternal, Newborn & Child Health reviewed evidence for the impact of different interventions and recommended a list of essential interventions for scale-up and the level of care where they can be provided, including several for newborns that can be delivered at community level: promotion and provision of thermal care, early and exclusive breastfeeding, and hygienic cord care [14].

From 2000 to 2012, Save the Children’s Saving Newborn Lives program (SNL) supported ministries of health in Bangladesh, Malawi, and Nepal to develop or strengthen national newborn programs. These programs include packages of maternal and newborn health interventions delivered through home visits [15], [16], [17]. All three countries had high neonatal mortality rates (NMR) at the start of the millennium (above 30 per 1000 live births) and thus the potential to see a large impact from increasing the number of women and newborns receiving postnatal care. At the same time, many women delivered outside health facilities, especially in Nepal and Bangladesh, so there was need to bring care to the community, though facility delivery rates rapidly increased over the program period (Table 1). These countries also had cadres of community health workers (CHWs) delivering services under ministries of health or other government agencies, providing a platform for community-based care for mothers and newborns.

**Table 1:** Country context.

	Bangladesh		Malawi		Nepal	
	2000	2011	2000	2011	2000	2011
Neonatal Mortality Rate (NMR) per 1,000 live births [1]	39	26	39	27	39	27
% of Under 5 deaths that were neonatal [1]	46%	57%	24%	33%	47%	56%
Proportion of health facility births [36], [37], [38], [39], [40], [41]	8%	29%	56%	73%	9%	35%
Total fertility rate [42]	3.1	2.2	6.1	6.0	4.1	2.7

**Note:** Data for proportion of health facility births in Malawi is from 2010; all other data under the columns marked 2011 is from 2011.

SNL supported implementation of home visits in selected districts to demonstrate the feasibility of delivering community-based maternal and newborn care through existing government health systems. This paper examines coverage and content of postnatal home visits in these areas. We also assessed the strength of association between three factors - receipt of home visits during pregnancy, birth place, and birth notification - and receipt of an early postnatal home visit, while controlling for potential confounders. We believe understanding these associations can help improve program design and planning in order to reach more mothers and newborns.

## Methods

### Setting and Program Description

In each country, districts were selected for SNL-supported implementation in partnership with governments. Implementation areas were four rural unions in Faridpur District, Bangladesh with a population of 98,000 people; portions of three districts in Malawi (Chitipa, Dowa, Thyolo) with a population of 711,000; and Bardiya District in the Terai area of Nepal with a population of 460,000.

Programs were designed to fit into health systems and used existing government authorized and trained workers or volunteers, supervisory and monitoring systems, facilities, and equipment and supplies [18], [19], [20]. SNL and other partners provided training in maternal and newborn care, add-on supplies, and support for supervision, monitoring, and community engagement.

The existing community cadres were all providing some care to mothers or children, but there were important differences across countries in terms of variations in their characteristics and how they were incentivized, outlined in Table 2. In Bangladesh, three cadres of community workers were trained to deliver home visits for women and newborns; all three were actively delivering health services under different government programs. Community workers in Bangladesh and Malawi were government salaried employees or volunteers paid regular stipends, while Nepal used volunteers incentivized with a performance-based scheme [21]. In all countries, CHWs were supposed to live in their catchment areas, and they were recruited from communities in Bangladesh and Nepal. However, community workers in Malawi were assigned posts by District Health Teams and only 47% interviewed in pilot districts reported living in their catchment areas [22].

**Table 2:** Community worker characteristics and home visit schedule, content, and incentives.

	Bangladesh	Malawi	Nepal
**Cadre name**	Family Welfare Assistant (FWA), Health Assistant (HA) - Females only, Community Nutrition Promoter (CNP)	Health Surveillance Assistant (HSA)	Female Community Health Volunteer (FCHV)
**Gender**	Female	Mostly male	Female
**CHW: pop. ratio**	FWA/HA - 1∶6000–7000, CNP - 1∶1250	1∶1000–2000	1∶400^1^
**Education level**	FWA/HA - Secondary, CNP - Primary(not strict)	Secondary	Literate, primary preferred
**Employment status**	FWA/HA - Govt salaried employee, CNP - Volunteer(stipend)	Govt salaried employee	Volunteer
**Recruitment**	Recruited from communities	Recruited centrally	Recruited from communities
**Pre-service training**	FWA - 21 days, HA - 6weeks, CNP - 24days	12 week	18 days
**Training in maternal newborn package**	5 days	9 days (+6 day Comm. Mobil.)	6 days
**Pregnancy visits**	2 (2^nd^ & 3^rd^ trimester)	3 (1^st^, 2^nd^ & 3^rd^ trimester)	4 (no specified timing)
**Content of pregnancy** **visits**	Encourage routine antenatal care and facility delivery		
	Promote birth preparedness including identifying facility/birth attendant, planning transport to facility, and saving money		
	Counsel on recognition of danger signs for mother and newborn and care-seeking		
	Promote newborn care including drying/wrapping, skin-to-skin contact, delayed bathing, immediate and exclusive breastfeeding, clean cord care		
	Promote optimal care for mother (breastfeeding, nutrition, family planning)		
	Counsel family to notify CHW (and other skilled birth attendant, if needed) at time of labor/delivery or immediately after delivery		
**Postnatal visits**	Day 1, 2–3, 4–7	Day 1 (home births), 3, 8	Day 1, 3, 7, 29
**Content of postnatal** **visits**	Reinforce newborn care messages and assessment of breastfeeding including support for feeding difficulties		
	Promote optimal care for mother (breastfeeding, nutrition, family planning)		
	Counsel on recognition of danger signs for mother and newborn and care-seeking		
	Screen for newborn danger signs/illness (examine baby, weigh baby, check temperature, check breathing).		
	First dose treatment with oral antibiotics for presumed serious bacterial infection (Nepal only)		
	Refer sick newborns		
	Counsel on extra care for low birth weight babies		
	Encourage routine facility or outreach care		
**Paid incentives based** **on number of visits**	No	No	Yes

1 Catchment area population size varies in Nepal depending on terrain; 400 population is based on Terai region such as Bardiya.

For the new programs, CHWs received in-service training on maternal and newborn care, ranging from five to nine days. CHWs were trained to identify pregnancies, visit women during pregnancy, and make three or four postnatal home visits, including a visit on the first day after birth (only for home births in Malawi) and two more visits within the first seven or eight days. Home visit schedules and content are shown in Table 2. Approximately 100 CHWs in Bangladesh, 600 in Malawi, and 850 in Nepal were trained in pilot areas.

In all countries, programs aimed to strengthen CHWs’ capacity to identify pregnancies through training and supervision. In Nepal, Family Community Health Volunteers (FCHVs) identified pregnant women during existing mothers’ group meetings and household visits (visit frequency depended on needs identified by mothers’ groups). In Bangladesh, Family Welfare Assistants (FWAs) identified pregnant women during routine, monthly household visits (though visits often occurred less frequently) and during regular outreach clinic sessions (each FWA has about 8 per month). Health Assistants (HAs) and Community Nutrition Promoters (CNPs) identified pregnant women during routine activities, such as family planning counseling. In Malawi, Health Surveillance Assistants (HSAs) made lists of women of child bearing age and were instructed to update the list every two months to identify pregnancies. Community “core groups” were also encouraged to report pregnancies to HSAs.

To facilitate early postnatal visits, pregnancy visits included counseling on when and how to notify the CHW about the birth. In Nepal, FCHVs were trained to attend home deliveries or accompany women to facilities, so pregnant women were instructed to call the FCHV at onset of labor. The Bangladesh program designed a mobile phone birth notification system and CHWs’ phone numbers were written on Mother’s Cards kept by families; families were instructed to notify CHWs after delivery. In Malawi, HSAs were instructed to encourage families to notify them after delivery, but counseling did not include specific notification instructions.

Content of home visits was similar across countries and included promotion of optimal maternal and newborn care and routine facility services, counseling on danger signs and care-seeking for mother and baby, screening for newborn danger signs by a physical assessment including checking the baby’s temperature and breathing and weighing the baby (if not previously weighed at a facility in Malawi and Nepal), and referral to a health facility when needed. In Nepal, some curative care was included; FCHVs administered an oral antibiotic for danger signs suggesting possible severe bacterial infection and referred the baby to health posts for a seven day course of injectable antibiotic.

CHWs were equipped with counseling cards, thermometers, scales, registers to record visits, and other supplies. In Nepal, FCHVs were given co-trimoxazole to treat suspected infection and a bag and mask and DeeLee suction to resuscitate babies that did not breathe immediately after birth. Add-on supplies were provided by SNL or partners.

In all countries, CHW supervisors received training to oversee home visits. In addition, SNL staff were involved in supervision visits, done in partnership with MOH staff when possible, to monitor implementation progress and identify gaps or problems. Program monitoring used existing reporting systems with the addition of new tools to collect maternal and newborn data. In Bangladesh, micro-planning meetings coordinated different CHW cadres by analyzing performance and mapping pregnancies to share home visit responsibilities.

In addition, programs included efforts to increase community engagement and support. In Bangladesh, community leaders attended orientation sessions to learn about the program. In Malawi, HSAs organized “core groups” for planning and decision-making. In Nepal, FCHVs discussed maternal and newborn health with existing mothers’ groups.

Start of full implementation to final data collection was 14 months in Bangladesh (April 2009–June 2010), 12 months in Malawi (June 2010–June 2011), and 17 months in Nepal (January 2010–June 2011). Home visits may have started earlier in some areas, depending when training was conducted.

### Data Sources

Data were from cross-sectional household surveys of women with a live birth in the previous 12 months (3–12 months in Bangladesh). Sample size calculations were based on expected changes in key indicators. In Bangladesh, pilot unions were divided into 12 clusters; six were selected randomly. Using probability proportionate to size (PPS) procedures, live births were randomly selected from lists of all live births collected prior to the survey. In Malawi, thirty clusters in each district were selected proportional to size using the 2008 population census (sampling restricted to villages where the program was implemented). Clusters were divided into segments, one segment selected, all houses numbered within the segment, and an index house randomly selected. Eligible women (age 15–49 with a live birth within the last 12 months) were interviewed. The next closest household was visited until 10 eligible women were interviewed per cluster. In Nepal, thirty clusters were selected proportional to size using the 2001 population census (excluding urban municipalities). Household lists were developed with key informants; the first household was selected randomly and subsequent households identified by spinning a bottle and all households within 20 meters of either side of a straight line were included until 21 eligible women (had a birth in the last 12 months) were interviewed. Only Nepal interviewed women with stillbirths; these cases were excluded from analysis. Response rates were 90% in Bangladesh, 100% in Malawi, and 91% in Nepal. Data were collected by teams of trained interviewers, and each team had a data quality supervisor. Data can be made available upon request.

### Variable Descriptions

The objective of this analysis was to examine the strength of association between three factors of programmatic interest –mother received a home visit during pregnancy, birth place, and CHW notified of the birth – and receipt of postnatal home visits from CHWs.

#### Dependent variable

The dependent variable was receipt of a postnatal home visit within three days after birth from a CHW. In Bangladesh and Malawi, women were asked about home visits before and after birth. In Nepal, women were not asked specifically about postnatal home visits. Instead, the woman was asked if she and the newborn were checked before discharge (or before the birth attendant left after a home birth), along with questions about the location and provider of the first two post-discharge checks on her health and the first three post-discharge checks on the newborn’s health. Since the woman was asked about only the first two post-discharge checks on her health, the percentage of women visited at home within three days after the birth appears lower compared to the percentage of newborns visited (41.3% versus 49.6%). Therefore, questions on post-discharge care for the baby were used to calculate the dependent variable in Nepal.

A visit occurred within three days if it was reportedly done within 72 hours after birth or on day 0, 1, 2, or 3. Currently, the global indicator for postnatal care includes care received within two days from any provider [5], [23]. However, this analysis focuses on home visits, and national policy in Malawi requires a postnatal home visit within three days for facility births. We used a visit within three days in all countries for comparability. The dependent variable was measured dichotomously, where 0 indicated no postnatal home visit within three days from a CHW and 1 indicated receipt of a visit.

#### Independent variables

The main independent variables, measured dichotomously, were defined as receipt of at least one home visit from a CHW during pregnancy (yes/no), place of birth (facility/non-facility), and whether a CHW was notified of the birth (yes/no). Questions on birth notification varied due to programmatic differences. In Bangladesh and Malawi, birth notification was based on whether women reported the CHW was notified of the birth. Since we examine the association between birth notification and receipt of home visits within 3 days, we classified birth notification as ‘no’ if the mother reported the CHW was notified more than 3 days after the birth. In Nepal, FCHVs were expected to attend deliveries, so birth notification was based on whether women reported that an FCHV was called at onset of labor. All analyses controlled for maternal age (<20, 20–29, 30+) and maternal education (any vs. none). Maternal age was made into a categorical variable because there was not a linear relationship between age and the log risk of receipt of a postnatal visit.

The newborn content of postnatal home visits was also analyzed, using standard metrics [24]: 1) checked the cord, 2) counseled on breastfeeding (including demonstration or observation), 3) checked the baby’s temperature, 4) weighed the baby, and 5) counseled on newborn danger signs. Questions about maternal content were not consistent across countries, and women in Nepal were only asked what was done for her health during the first post-discharge check. In addition, content questions were prompted in Malawi and Nepal, but unprompted in Bangladesh. For comparability, only newborn content data from Malawi and Nepal are presented.

### Statistical Analyses

Descriptive statistics were used to describe characteristics of interviewed women in the three countries. Pairwise correlation and collinearity among variables were evaluated. Generalized linear models were used to assess the relationship between the three primary independent variables – mother received a home visit during pregnancy, birth place, and birth notification – and receipt of a postnatal home visit within three days. Because data were collected within communities (i.e. clusters), not accounting for clustering may lead to incorrect statistical inference, such as underestimated standard errors and biased point estimates [25], [26]. We thus controlled for clustering in all analyses and appropriate standard error estimates were produced using the Taylor linearization method [27]. Relative risks (RR) and 95% confidence intervals (CI) were obtained.

To understand whether similar trends were seen across countries, we generated adjusted pooled relative risk estimates of each primary independent variable on the main outcome. We used meta-analytic techniques, and pooled estimates were adjusted for maternal age and education as well as the other primary independent variables. The pooled relative risk estimates the average weighted association between the main outcome and each of the three independent variables [28]. We fitted both fixed effect models using the inverse-variance fixed-effect method and random effects models using the DerSimonian and Laird method to determine if there was heterogeneity among countries; that is, whether the true relationship between exposures of interest and the likelihood of a postnatal home visit is not the same in each country [29]. The resulting *I^2^* statistic, the percentage of between-study heterogeneity attributable to variability in the true relationship, and the heterogeneity chi-squared test indicated there was statistically significant heterogeneity between countries for the relationship between birth notification and postnatal home visits (p = 0.000), so the fixed effect model was inappropriate for this case. There was no heterogeneity between countries for the relationship between pregnancy home visits or birth place and postnatal home visits, and the summary statistic and confidence intervals were the same whether fixed or random effects models were used. Therefore, we report results using the random effects models. We also did a sensitivity analysis for birth notification. Such meta-analyses have been used in the literature of cross-sectional studies [28]. STATA 11.0 was used for all analyses [30].

### Ethics Statement

Programs were implementing national policy through routine systems. Ethical clearance was obtained from the Bangladesh Medical and Research Council and the National Health Sciences Research Committee in Malawi. Per approved protocols, women gave oral consent to participate in surveys due to high levels of illiteracy. To operationalize the National Neonatal Health Strategy [31], the Nepal Ministry of Health and Planning initiated the development of the Community-Based Newborn Care Package, which outlined the role of Save the Children in supporting the government to develop and test the package [32]. Data collection was completed as part of routine programmatic activities. Relevant district authorities granted permission and all respondents provided oral consent upon being informed of the purpose of data collection. Consent was documented by interviewers on the questionnaires.

## Results

The number of interviewed women with live births was 398 in Bangladesh, 900 in Malawi, and 615 in Nepal. Table 3 shows the distribution of independent variables. Nearly all women in Bangladesh and Nepal reported at least one home visit during pregnancy from a CHW (90% and 97%, respectively), while only 36% of women in Malawi reported a pregnancy visit. Facility birth rates were highest in Malawi (92%), followed by Nepal (81%), and were much lower in Bangladesh (26%). In Malawi and Nepal, these rates were higher than the 2010 national average, though baseline program data indicates these areas had average rates at the start of implementation [33], [34], [35]. Reported birth notification was highest in Nepal (66%), followed by Bangladesh (40%), then Malawi (20%).

**Table 3:** Distribution of independent variables^1^.

	Bangladesh (N = 398)	Malawi (N = 900)	Nepal (N = 615)
**Pregnancy home visit (≥1)**			
No	9%	63%	3%
Yes	90%	36%	97%
**Place of birth**			
Facility	26%	92%	81%
Non-facility	74%	7%	19%
**Birth notification within 3 days**			
No	60%	79%	34%
Yes	40%	20%	66%
**Maternal age**			
<20	14%	15%	15%
20–29	65%	56%	74%
30+	21%	27%	11%
**Maternal education**			
No education	16%	12%	42%
Any education	84%	87%	58%

1 Missing values were <3% for all variables.

### Postnatal Home Visit Coverage and Content

**Figure 1:**
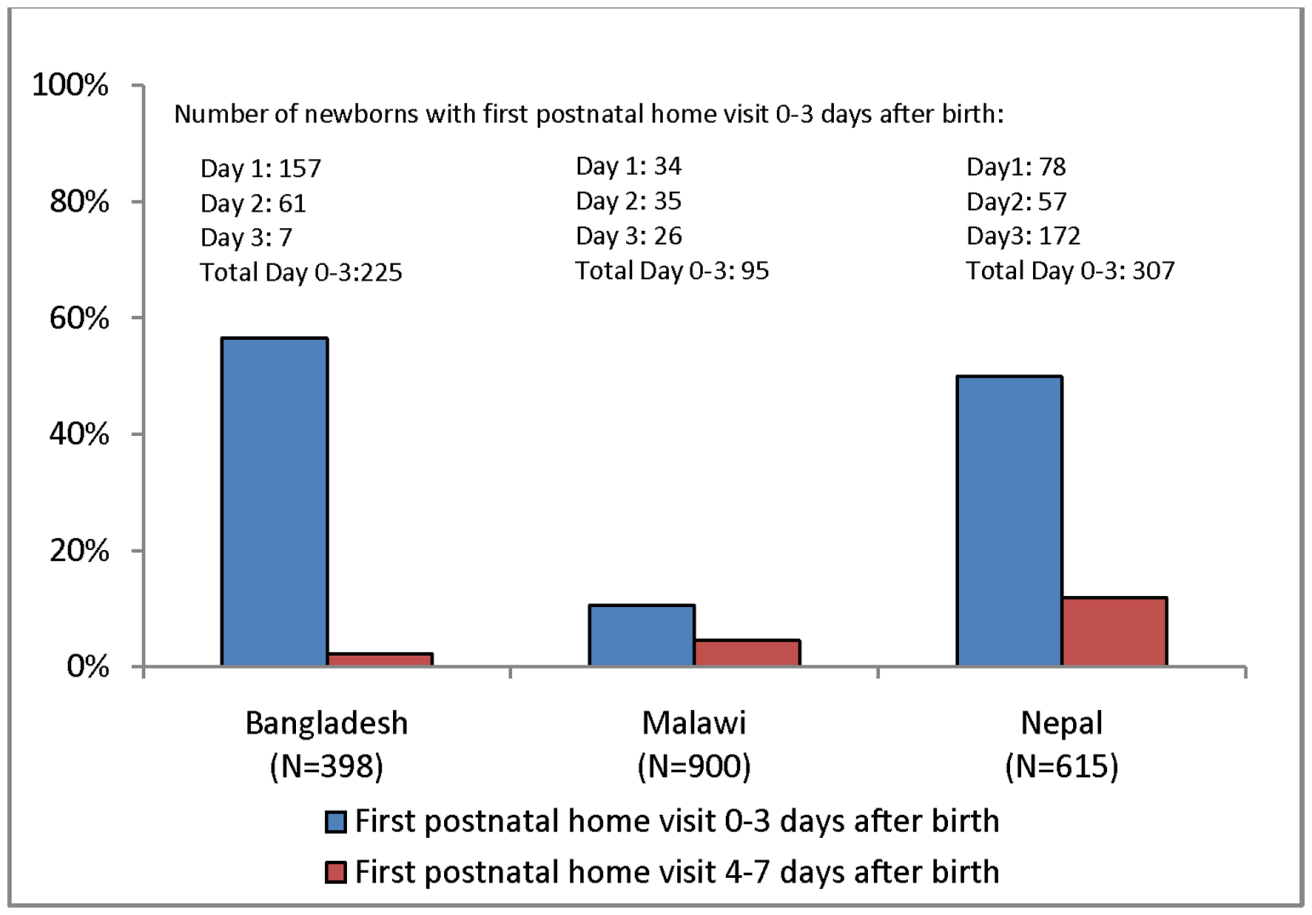
Proportion of mothers^1^ and newborns receiving CHW home visits in the first week after birth. This figure shows the percent of mothers and newborns that received a home visit from a community health worker within 0–3 days after birth and 4–7 days after birth in each of the 3 countries included in the analysis – Bangladesh, Malawi, and Nepal. ^1^In Nepal, separate questions were asked about postnatal care for the mother and newborn. The woman was asked about only the first two post-discharge checks on her health, but was asked about the first three post-discharge checks for her newborn. Thus the percentage of women visited at home within three days after the birth appears lower than the percentage of newborns visited (41.3% versus 49.6%). Therefore, questions on post-discharge care for the baby were used to calculate the dependent variable in Nepal.

The proportion of mothers and newborns who received a home visit from a CHW within three days after birth was 57% in Bangladesh, 11% in Malawi, and 50% in Nepal. Figure 1 shows the proportion of mothers and newborns who received an initial postnatal home visit within three days after birth and four to seven days after birth. If the mother and newborn received a home visit during the first week after birth, the first visit was usually done within three days.

Table 4 shows newborn care actions carried out by CHWs for newborns who received a visit within three days after birth in Malawi and Nepal. Every action was reported for at least 65% of these newborns, with the exception of weighing the baby in Nepal. In both countries, checking the cord was the most frequently reported action; nearly all women reported at least one action.

**Table 4:** Newborn care content of postnatal home visits within 3 days after delivery.

Actions.	Malawi^1^		Nepal	
	n	%	n	%
Checked the cord	85	90%	287	94%
Counseling on breastfeeding^2^	83	87%	240	78%
Checked temperature	78	82%	200	65%
Weigh the baby	82	86%	94	31%
Counseling on danger signs	66	70%	–	–
At least 1 of 4 common actions^3^ reported	93	98%	307	100%
All 4 common actions reported	67	71%	69	23%
*TOTAL*	*95*		*307*	

1 In Malawi, women were asked what was done by an HSA during any home visit; it was assumed that all reported actions applied to visits that occurred within 3 days of delivery for newborns that received multiple visits.

2 Counseling on breastfeeding included observation, demonstration, or assessment of breastfeeding.

3 Check the cord, counsel on breastfeeding, check temperature, and weigh baby were collected in both countries. Counseling on danger signs is excluded since it was only collected in Malawi.

### Strength of Association between Receipt of Postnatal Home Visits and Key Factors

Table 5 shows the crude and adjusted relative risk of receiving a home postnatal visit within three days after birth in each country and the adjusted pooled relative risk.

**Table 5:** Relative risk for receiving a postnatal home visit within 3 days after birth.

	Bangladesh				Malawi				Nepal				Meta-Analysis	
		Relative risk				Relative risk				Relative risk			Relative risk	
	n	Crude	Adjusted (95% CI)	p value	n	Crude	Adjusted(95% CI)	p value	n	Crude	Adjusted (95% CI)	p value	Adjusted (95% CI)	p value
**Pregnancy home visits**														
None (Ref)	39				520				18					
At least one	359	2.95	2.45 (1.29–4.68)	0.008	278	7.37	2.22 (1.16–4.27)	0.017	357	1.82	1.61 (0.63 –4.16)	0.309	2.18 (1.46–3.25)	0.000
**Place of delivery**														
Facility (Ref)	105				745				501		1.00			
Non-facility	293	1.66	1.35 (1.02–1.79)	0.038	53	1.69	1.24(0.82–1.89)	0.303	114	1.45	1.63 (1.32–2.00)	0.000	1.48 (1.28–1.73)	0.000
**Birth notification**														
No (Ref)	240				644				207		1.00			
Yes	158	1.97	1.68 (1.42–1.99)	0.000	154	17.77	12.01 (5.63–25.59)	0.000	408	1.37	1.49(1.13–1.96)	0.006	2.66 (1.40–5.08)	0.003
**Maternal age**														
<20 (Ref)	55				118				96		1.00			
20–29	257	1.26	1.23 (0.92–1.64)	0.150	458	0.85	0.86 (0.60–1.23)	0.392	453	1.30	1.29 (0.97–1.72)	0.075	NA	NA
30+	86	1.36	1.19 (0.84–1.69)	0.313	222	0.91	0.94 (0.60–1.47)	0.768	66	1.08	1.08 (0.70–1.64)	0.728		
**Maternal Education**														
None (Ref)	65				92				258					
Any	333	0.88	0.89 (0.69–1.15)	0.378	706	1.43	1.72 (0.94–3.18)	0.080	357	1.02	1.06 (0.90–1.24)	0.487	NA	NA
*TOTAL*	*398*				*798*				*615*					

#### Home visits during pregnancy

Among women receiving at least one home visit during pregnancy in Bangladesh and Nepal, a small proportion received just one visit (10% in Bangladesh, 3% in Nepal) while most women received four or more (57% in Bangladesh, 63% in Nepal). In Malawi, many women received just one home visit (39%) or two to three visits (51%), and very few received four or more (6%).

Mothers and newborns were more likely to have received a postnatal home visit within three days after birth if the mother received at least one home visit during pregnancy in Bangladesh (RR2.45, CI1.29–4.68) and Malawi (RR2.22, CI1.16–4.27). There was no statistically significant difference in Nepal, where almost all women received at least one pregnancy home visit. The cross country meta-analysis summary estimate showed mothers and newborns were two times more likely to receive a postnatal visit within three days if the mother received at least one home visit during pregnancy (RR2.18, CI1.46–3.25).

#### Place of delivery

Mothers and newborns were more likely to receive a postnatal home visit within three days if the birth occurred outside a facility in Bangladesh (RR1.35, CI1.02–1.79) and Nepal (RR1.63, CI1.32–2.00). The same trend was seen in Malawi but was not statistically significant. The meta-analysis summary estimate showed mothers and newborns delivered outside facilities were 48% more likely to receive a home visit within three days after birth (RR1.48, CI1.28–1.73).

#### Birth notification

Women in Bangladesh and Malawi were asked who notified a CHW of the birth, how, and when. Husbands or other family members most commonly notified the CHW (80% in Bangladesh, 76% in Malawi) while few notifications were reportedly done by delivery attendants (9% in Bangladesh, 2% in Malawi) or others (12% in Bangladesh, 10% in Malawi). Notification was most often done by visiting the CHW (49% in Bangladesh, 64% in Malawi). Mobile phones were also commonly used in Bangladesh (33%). Most birth notifications were done within three days after birth; this proportion was higher in Bangladesh than in Malawi (82% vs. 63% of births notified). In Nepal, families were instructed to notify FCHVs at onset of labor, but 34% did not; the most common reasons given for not notifying an FCHV were delivery occurred at night (46%), the FCHV was out of the village (19%), and it was felt to be unnecessary (16%).

In all countries, mothers and newborns were more likely to receive a home visit within three days after birth if the mother reported a CHW was notified of the birth. This association was particularly dramatic in Malawi (RR12.01, CI5.63–25.59). The meta-analysis summary estimate showed mothers and newborns were over two times more likely to receive a postnatal home visit within three days when mothers reported that birth notification took place (RR2.66, CI1.40–5.08).

We also ran a sensitivity analysis removing data from Malawi. The resulting summary statistic was in the same direction (RR1.63, CI1.42–1.87), indicating the effect was not solely driven by Malawi where the association was strongest.

#### Maternal age and education

In all countries, maternal age and education had no statistically significant association with postnatal home visits within three days.

## Discussion

This paper presents coverage and content of postnatal home visits delivered by very different cadres of community-based workers in three low-income, high neonatal mortality countries. Maternal-newborn home visit packages are relatively recent in national policies and most existing analyses are from trials. To our knowledge, this is the first multi-country analysis of delivery of postnatal home visits using routine national systems that attempted to understand factors associated with receipt of home-based postnatal care.

The postnatal home visit approach was implemented in pilot areas using existing government systems and staff (or volunteers), with inputs where necessary of essential equipment and supplies and resources to support training, supervision, and monitoring. The proportion of mothers and newborns who received a home visit within three days after birth was highest in pilot areas of Bangladesh at 57% and lowest in Malawi at 11%. If a home visit occurred during the first week after birth, it usually occurred during the first three days. Increasing coverage of early postnatal home visits will require strategies to reach those newborns that would otherwise be missed.

One or more home visits during pregnancy increased the likelihood of receiving an early postnatal visit. Pregnancy visits provide opportunities for counseling on birth preparedness and the importance of postnatal care and allow CHWs to develop relationships with families. The proportion of mothers receiving pregnancy visits was lower in Malawi, where HSAs often do not live in communities they serve and have a diverse set of responsibilities such as water and sanitation, integrated community case management, and tuberculosis and HIV care. Home visits to pregnant women may be challenging to implement in settings where pregnancy surveillance is weak or where CHWs do not reside in their catchment areas, have large catchment areas, or are assigned many tasks. Where it is difficult for CHWs to actively identify pregnancies, strategies are needed to engage families, communities, and facility providers to notify CHWs. For example, community or women’s groups could be encouraged to inform CHWs of pregnancies and facility-based providers could notify CHWs when women attend their first routine ANC visit.

Though mothers that give birth at facilities need pre-discharge postnatal care for themselves and their newborns, they also need continued care in the first week after discharge. Yet facility births were less likely to receive early postnatal care at home. Increasing coverage of postnatal home visits for facility births can ensure continuum of care from facility to home, and can be done by strengthening linkages between facilities and CHWs to ensure CHWs are informed when babies are discharged. Where CHWs are overstretched and it is difficult to reach all mothers and newborns, facility staff could notify CHWs of high risk newborns at discharge so they can target postnatal visits to the most vulnerable for the greatest impact.

A very small proportion of babies born outside facilities received facility-based postnatal care within three days after birth (3% in Bangladesh, 10% in Malawi, 6% in Nepal), so home visits remain a crucial service for this subset of newborns and a mechanism to promote care-seeking from skilled providers at health facilities.

Timely birth notification of CHWs appears to be important for ensuring early postnatal home visits. Household interviews may have underestimated birth notification if done without the mother’s knowledge or have overestimated the importance of notification if mothers wrongly assumed notification took place or if women who reported a visit within three days were more likely to report notifying a CHW. Birth notification was most strongly associated with early postnatal visits in Malawi, where CHWs usually reside outside the community; in this context, active notification by the family may have been the only way CHWs were able to learn of births, especially since facility staff were not engaged in notifying CHWs. Birth notification strategies should be tailored to the local context and may include use of mobile phones; raising awareness of the importance of birth notification and postnatal home visits among families, CHWs, and health facility staff; and regular planning and close coordination between CHWs and supervisors based at facilities. Engaging facility staff in birth notification will be especially important in settings with high or increasing facility delivery rates.

Reported actions by CHWs during postnatal home visits were analyzed as markers for quality of care. Five actions were considered, all related to newborn care, and results were high, with the exception of weighing the baby in Nepal. FCHVs are only expected to weigh newborns delivered at home, and high facility delivery rates may explain the low proportion weighed during home visits. These data suggest that CHWs are able to conduct basic postnatal care tasks in settings where they receive training, supplies, and supervision. Still, quality of postnatal care is an area that requires careful monitoring.

In addition to factors examined in our analyses, the proportion of mothers and newborns that receive home visits depends on availability and motivation of trained CHWs, which are influenced by many conditions. The proportion receiving visits was higher in Bangladesh and Nepal, where CHWs were locally recruited and resident in their catchment areas. FCHVs in Nepal had relatively small catchment populations. In addition, FCHVs were able to give a first dose oral antibiotic, which may have increased the value of home visits for families and thus demand or increased motivation of FCHVs because they felt they had something of value to offer families. FWAs and HAs in Bangladesh have large catchment populations, but may face fewer geographic barriers due to high population density and another category of CHW serving the same areas (CNPs) were trained to complement their work. Effective coverage of a scaled up program in Bangladesh will vary depending on the number of trained CHWs available and their workload. The proportion of mothers and newborns visited was lower in Malawi where HSAs were centrally recruited, often did not reside in communities, had large catchment populations, and were expected to spend at least two days per week at a facility and fulfill multiple responsibilities. All workers received some compensation, though the type varied. CHW effectiveness is also influenced by the quality and frequency of supervision and availability of equipment and supplies. The importance of CHW gender may be quite different depending on local cultural norms, but should be investigated and understood during program planning. All these factors need to be considered when designing an implementation plan for postnatal home visits. In the three countries described here, systems for selection and deployment of CHWs were already developed and postnatal home visits were added to existing responsibilities. It is recognized that using established systems and adding new tasks creates challenges, but program strategies can be adapted and existing systems can also be modified to accommodate changing program priorities.

Analytic limitations should be noted. Household survey questionnaires differed between countries; variations that may affect comparability have been identified. Questionnaires were administered to women with a live birth in the previous year, which may have introduced recall or reporting bias.

### Conclusions

Results from pilot implementation areas in Bangladesh, Malawi and Nepal demonstrate that reaching mothers and babies with home visits during pregnancy and within three days after birth is achievable using existing community health systems if workers are available; linked to communities; and receive training, supplies, and supervision. Programs can increase the reach of postnatal visits with approaches that support CHWs to identify and visit pregnant women and ensure that CHWs are notified of home and facility births. These factors were important in all three countries, despite program differences, and are likely to be important in all settings where postnatal home visits are introduced into local health systems. Still, regardless of the inputs, many women and newborns were not reached at home soon after birth. In all settings, programs must evaluate what community delivery systems can handle, how to best utilize them to improve postnatal care access, and what other aspects of the health system can be strengthened to improve outcomes for mothers and newborns.
